# Using Different Single-Step Strategies to Improve the Efficiency of Genomic Prediction on Body Measurement Traits in Pig

**DOI:** 10.3389/fgene.2018.00730

**Published:** 2019-01-14

**Authors:** Hailiang Song, Jinxin Zhang, Qin Zhang, Xiangdong Ding

**Affiliations:** ^1^Key Laboratory of Animal Genetics and Breeding of Ministry of Agriculture, National Engineering Laboratory of Animal Breeding, College of Animal Science and Technology, China Agricultural University, Beijing, China; ^2^Shandong Provincial Key Laboratory of Animal Biotechnology and Disease Control and Prevention, Shandong Agricultural University, Taian, China

**Keywords:** body measurement traits, pig, single-step GBLUP, two-trait model, cross-validation

## Abstract

In genomic prediction, single-step method has been demonstrated to outperform multi-step methods. This study investigated the efficiency of genomic prediction for seven body measurement traits in Yorkshire population and simulated data using single-step method. For Yorkshire population, in total, 592 individuals were genotyped with Illumina PorcineSNP80 marker panel. We compared the prediction accuracy obtained from a traditional pedigree-based method (BLUP), a genomic BLUP (GBLUP) and a single-step genomic BLUP (ssGBLUP) through 20 replicates of 5-fold cross-validation (CV). In addition, we also compared the performance of two-trait ssGBLUP and single-trait ssGBLUP for the traits with different gradients of genetic correlation. Our results indicated the GBLUP method generally provided lower accuracies of prediction than BLUP and ssGBLUP methods, and the average standard deviation of unbiasedness was as large as 0.278. For single-step methods, the accuracies of ssGBLUP for seven body measurement traits ranged from 0.543 to 0.785, and the unbiasedness of ssGBLUP ranged from 0.834 to 1.026, respectively. ssGBLUP generally generated 1% on average higher prediction accuracy than traditional BLUP, the improvement of ssGBLUP and the performance of GBLUP was lower than expected mainly due to the small number of genotyped animals, it was further demonstrated by our simulation study. We simulated two traits with heritabilities 0.1, 0.3, and with high genetic correlation 0.7, our results also showed that the prediction accuracies were low for GBLUP compared with other three methods with different genotyped reference population sizes and the accuracies were improved with increasing the genotyped reference population size. However, the increase was small for ssGBLUP compared with BLUP when the genotyped reference population size was <500. Our results also demonstrated that the accuracies of genomic prediction can be further improved by implementing two-trait ssGBLUP model, the maximum gain on accuracy was 2 and 2.6% for trait of chest width compared to single-trait ssGBLUP and traditional BLUP, while the gain was decreased with the weakness of genetic correlation. Two-trait ssGBLUP even performed worse than single trait analysis in the scenario of low genetic correlation.

## Introduction

The purpose of animal breeding is to genetically improve the performance of a population, which is usually achieved by selecting the best animals among the current generation to be served as parents of the next generation. In pig breeding, the main objective traits are growth and reproduction traits, such as days to 100 kg, backfat thickness and total number of piglets born. Body measurement traits are usually not concerned because of the difficulty of phenotypic data collection and its economic importance. However, the genetic evaluation of body measurement traits is becoming more important recently. The estimation of live weight by body measurements have been applied to different animal species (Enevoldsen and Kristensen, 1997; Thiruvenkadan, 2005). And the accuracy of weight prediction using body length and heart girth for pigs from different age categories had been reported by Mutua et al. (2011).

Since the historic work of Meuwissen et al. (2001), combining genome data with corresponding statistical models has been successfully applied to genome selection. The key issue of genomic selection is to predict individual genomic breeding values (GEBV) using genome-wide marker information. Many statistical methods have been developed to predict GEBV, which are basically different in the assumption of distribution of SNP effects. The linear BLUP models (at either the SNP level or the individual animal level) assume that effects of all SNP are normally distributed with same variance (Meuwissen et al., 2001; VanRaden, 2008). On the other hand, the Bayesian Alphabet methods (e.g., BayesA, BayesB, and BayesCpi) (Meuwissen et al., 2001; Habier et al., 2011) allow each SNP effect to have its own variance. Many studies have reported that Bayesian methods performed similar to genomic BLUP (GBLUP) model in real data (Hayes et al., 2009a) and GBLUP is also simpler and lower computation-demanding than the Bayesian Alphabet methods.

Generally, genomic prediction utilizes information of genotyped animals. In practice, however, only a subset of individuals can be genotyped. Furthermore, in order to make use of phenotype information of non-genotyped individuals, a single-step GBLUP (ssGBLUP) has been developed by constructing H matrix using marker genotypes and pedigree jointly instead of G matrix or pedigree-based relationship matrix alone (Legarra et al., 2009; Christensen and Lund, 2010). Field data of cattle, pigs and chickens indicated that single-step method leads to higher accuracy and much simpler than multi-step genomic selection methods (Aguilar et al., 2011; Chen et al., 2011; Forni et al., 2011; Christensen et al., 2012; Simeone et al., 2012; Li et al., 2014; Song et al., 2017).

Genomic selection usually handles a single trait only. However, many traits are genetically correlated. As in traditional genetic evaluation, a multi-trait model is expected to increase the accuracy of the GEBV by making use of information from genetically correlated traits which will be more profound for traits with low heritability or with a small number of phenotypic records (Jia and Jannink, 2012; Guo et al., 2014). Many studies report multi-trait model for genetically correlated traits could lead to more accurate predictions than single trait genomic prediction (Calus and Veerkamp, 2011; Jia and Jannink, 2012; Guo et al., 2014; Wang et al., 2017).

The objective of this study was to: (1) estimate genetic parameters of seven body measurement traits; (2) assess the accuracy of genomic selection using single-step strategy in the scenario of small genotyped animals; (3) improve the accuracy of genomic prediction using two-trait ssGBLUP with different gradients of genetic correlation; (4) investigate the impact of different genotyped reference population sizes on the accuracy of genomic prediction using simulated data.

## Materials and Methods

### Ethics Statement

The whole procedure for collecting pig blood samples was carried out in strict accordance with the protocol approved by the Animal Care and Use Committee of China Agricultural University (Permit Number: DK996).

### Simulated Data

The multiple-trait genomic simulation software GPOPSIM (Zhang et al., 2015) was used to simulate the genomic data and phenotypic data. Trait A (h2 = 0.1) and trait B (h2 = 0.3) with high genetic correlation of 0.7 were simulated, the population included 30,000 individuals with 10 generations. We simulated 18 chromosomes with a total length of 18 Morgan, each chromosome included 2,834 markers which were evenly distributed, a total of 51,012 markers and 306 QTLs, and the mutation rates of markers and QTLs were 1.25 × 10^−3^ and 2.5 × 10^−3^, respectively. Mimicking the pig breeding, phenotypic values were generated by adding simulated phenotypes and fixed effect values, and the fixed effects include sex-generation which were generated through uniform distribution from 0 to 1. We randomly selected 1,000 individuals from generation 10 as validation population, genotyped reference populations of different sizes of 50, 100, 300, 500, 1,000, 2,000, and 3,000 were randomly sampled from generation 8, 30,519 animals were traced back to construct pedigree relationship matrix.

### Body Measurement Traits Data of Yorkshire

#### Population and Phenotypes

The data from an elite Chinese pig breeding farm which are descendant of American Yorkshire populations. The information was shown in Table 1, phenotypic records of body measurement traits included body length (BL), body height (BH), chest width (CW), rump width (RW), chest girth (CG), tube girth (TG), and abdominal girth (AG). The conventional estimated breeding value (EBV) were calculated based on a 7-trait animal model for the body measurement traits. The fixed effects include herd-year-season-sex. The random effects include additive genetic effect of each individual and random residual. Furthermore, body weight was taken into account as covariate, 7,020 animals were traced back to construct pedigree relationship matrix.

**Table 1:** Descriptive statistics of seven body measurement traits and two simulated traits.

**Trait**^**a**^	**N-obs**^**a**^	**Birth year**	**Average**	**SD**
BL	5,573	2013–2016	108.88	6.18
BH	5,573	2013–2016	62.87	2.92
CW	5,572	2013–2016	29.75	2.30
RW	5,573	2013–2016	31.64	2.13
CG	5,573	2013–2016	104.58	5.75
TG	5,573	2013–2016	17.98	1.03
AG	4,898	2013–2016	113.52	6.30
Trait A	30,000	–	2.25	3.13
Trait B	30,000	–	1.65	2.51

a *BL, body length; BH, body height; CW, chest width; RW, rump width; CG, chest girth; TG, tube girth; AG, abdominal girth; Trait A and Trait B were simulated traits with genetic correlation of 0.7*.

b *N-obs, number of observations*.

#### Genotype Data

Genomic DNA was extracted from blood sample using TIANamp Blood DNA Kit DP348 (Tiangen, Beijing, China). Genotyping was performed using a PorcineSNP80 BeadChip (Illumina, San Diego, CA, USA) which includes 68,528 SNPs across the entire pig genome. In total, 592 pigs with body measurement traits records were genotyped.

Missing genotypes of SNPs with known chromosomal positions were imputed by Beagle (Browning and Browning, 2009), and those with unknown position or on the X chromosomal were discarded from the study. SNPs loci with minor allele frequency < 0.05, call frequency score < 0.90 and Hardy-Weinberg equilibrium with a *P*-value < 10^−7^ were excluded, in addition, the individuals with reliability of EBV <0.35 for 7 body measurement traits were removed. After quality control, all genotyped individuals remained and 55,759 SNPs were finally used.

### Statistical Models

Four methods, a traditional BLUP method with pedigree–based relationship matrix, a GBLUP method based on genomic relationship matrix, a single-trait single step GBLUP (ssGBLUP) and two-trait ssGBLUP methods with combined relationship matrix constructed from marker and pedigree information, were used to predict breeding values.

#### BLUP

The traditional animal model with a pedigree-based relationship matrix was applied to predict breeding values for each trait separately. The model was defined as:

y=Xb+γW+Zg+e,

for real data of pig, where **y** is the vector of phenotypic values, **b** is the vector of fixed effects including herd-year-season-sex, **X** is the incidence matrix associating **b** with **y**, **W** is the covariate of body weight, **γ** is the regression coefficient, **g** is the vector of additive genetic effects, following a normal distribution of $N\left(0,\;\text{A}{\sigma_{g}}^{2}\right)$, in which **A** is the matrix of additive genetic relationships, ${\sigma_{g}}^{2}$ is the variance of additive genetic effect. **Z** is the incidence matrix associating **g** with **y**; **e** is the vector of random residuals with distribution of N(0, ${{\text{I}\sigma }_{e}}^{2}$), in which **I** is the identity and ${\sigma_{e}}^{2}$ is the residual variance. For simulated data, the model is the same as real data except **b** is the vector of fixed effects including sex-generation and no covariate.

#### GBLUP

GBLUP (VanRaden, 2008) model was used to predict GEBV of all genotyped individuals.

$$y_{c}=1u+Za+e,$$

where **y**_**c**_ is the vector of corrected phenotypic values which were computed as the EBV plus estimated residual for each individual for simulated and real data, **u** is the overall mean, **1** is a vector of 1, **a** is the vector of genomic breeding values, following a normal distribution of N(0, **G**${\sigma_{a}}^{2}$), in which ${\sigma_{a}}^{2}$ is the variance of addictive genetic effect and **G** is the marker-based genomic relationship matrix (VanRaden, 2008). **e** is the vector of random errors, following a normal distribution of N(0, **I**${\sigma_{e}}^{2}$), in which ${\sigma_{e}}^{2}$ is the residual variance.

#### Single-Trait ssGBLUP

The ssGBLUP model using information of both genotyped and non-genotyped phenotype information, and using both marker and pedigree information for genetic evaluations. The single-trait ssGBLUP has the same model as BLUP, except vector **g** is assumed to follow a normal distribution $N\left(0,\;\text{H}{\sigma_{g}}^{2}\right)$. Following Legarra et al. (2009), Christensen and Lund (2010), and Aguilar et al. (2011), the **H** was defined as:

$$H=\left[\begin{array}{cc} A_{11}+A_{12}A_{22}{}^{-1}\left(G_{w}-A_{22}\right)A_{22}{}^{-1}{A^{\prime }}_{12} & A_{12}A_{22}{}^{-1}G_{w}\\ G_{w}A_{22}{}^{-1}{A^{\prime }}_{12} & G_{w} \end{array}\right],$$

in which **A**_11_, **A**_12_, and **A**_22_ were the sub-matrices of **A** (the pedigree-based relationship matrix), and subscripts 1 and 2 refer to non-genotyped and genotyped animals, respectively. Compared with the H matrix, the inverse of H was simple which used to solve the mixed model equations, the inverse of H was:

$$H^{-1}=\left[\begin{array}{cc} G_{w}^{-1}-A_{22}^{-1} & 0\\ 0 & 0 \end{array}\right]+A^{-1}.$$

To avoid singularity problems, **G**_**w**_ **= 0.95G**_**a**_**+0. 05A**_**22**_ (Aguilar et al., 2010; Lourenco et al., 2014), **G**_**a**_ is an adjusted **G**, in order to avoid the differences in scale and location between the coefficients of **G** and pedigree relationship matrix (**A**_22_), the **G** matrix was adjusted according to Christensen et al. (2012),

$$\begin{array}{l} {\text{G}}_{a}=\text{G}\beta +\alpha , \end{array}$$

where α and β are adjustment factors derived from the following equations:

$$\begin{array}{l} \text{ Avg}.\text{diag}\left(G\right)\beta +\alpha =\text{Avg}.\text{diag}\left(A_{22}\right)\text{ and}\\ \text{Avg}.\text{offdiag}\left(G\right)\beta +\alpha =\text{Avg}.\text{offdiag}(A_{22}), \end{array}$$

in which Avg.diag is the average of the diagonal elements, and Avg.offdiag is the average of the off-diagonal elements.

#### Two-Trait ssGBLUP

The two-trait model was defined as:

$$\left[\begin{array}{c} y_{1}\\ y_{2} \end{array}\right]=\left[\begin{array}{cc} X_{1} & 0\\ 0 & X_{2} \end{array}\right]\left[\begin{array}{c} b_{1}\\ b_{2} \end{array}\right]+\left[\begin{array}{c} \gamma_{1}\\ \gamma_{2} \end{array}\right]\left[\begin{array}{c} W_{1}\\ W_{2} \end{array}\right]+\left[\begin{array}{cc} Z_{1} & 0\\ 0 & Z_{2} \end{array}\right]\left[\begin{array}{c} g_{1}\\ g_{2} \end{array}\right]+\left[\begin{array}{c} e_{1}\\ e_{2} \end{array}\right],$$

where $\left[\begin{array}{c} y_{1}\\ y_{2} \end{array}\right]$ is the vector of observation values of trait I and II, **b**_**1**_ and **b**_**2**_ are the vector of fixed effects of herd-year-season-sex for real data and sex-generation for simulated data of trait I and II, **X**_**1**_ and **X**_**2**_ are the incidence matrix associating **b**_**1**_ and **b**_**2**_ with **y**_**1**_ and **y**_**2**_, $\left[\begin{array}{c} w_{1}\\ w_{2} \end{array}\right]$ is the vector of covariate of body weight of trait I and II for real data and no covariate for simulated data, **γ**_**1**_ and **γ**_**2**_ are the regression coefficient associating **W**_**1**_ and **W**_**2**_, $\left[\begin{array}{c} g_{1}\\ g_{2} \end{array}\right]$ is the vector of additive genetic effects of the two traits, following a normal distribution of *N*(**0**, **H**⊗**M**), where **M** = $\left[\begin{array}{cc} \sigma_{\text{g}1}^{2} & \sigma_{\text{g}12}^{2}\\ \sigma_{\text{g}12}^{2} & \sigma_{\text{g}2}^{2} \end{array}\right]$ is the variance and covariance matrix of the genomic breeding values of the two traits, **Z**_**1**_ and **Z**_**2**_ are the incidence matrix associating **g**_**1**_ and **g**_**2**_ with **y**_**1**_ and **y**_**2**_, $\left[\begin{array}{c} e_{1}\\ e_{2} \end{array}\right]$ is the vector of random errors with distribution of *N*(**0**, **I⊗R**), where **I** is the identity matrix and R = $\left[\begin{array}{cc} \sigma_{e1}^{2} & \sigma_{e12}^{2}\\ \sigma_{e12}^{2} & \sigma_{e2}^{2} \end{array}\right]$is the residual variance and covariance matrix of the two traits.

### Evaluation of the Accuracy of Genomic Prediction

In this study, the accuracy and unbiasedness of prediction were obtained through 5-fold cross-validation (CV) for real data. The genotyped individuals were randomly partitioned into five nearly equal sized subpopulations. Of the five subpopulations, a single subpopulation was retained as the validation population and the remaining four subpopulations were used as reference population. The cross-validation process was then repeated five times, with each of the five subpopulations used exactly once as the validation population. For all scenarios, the 5-fold CV was replicated 20 times, resulting in 20 averaged accuracies of genomic prediction. The two-trait ssGBLUP with CV was also implemented to predict GEBV for the traits with different gradients of genetic correlation. For BLUP, GBLUP and ssGBLUP, the validation set was same in each replicate of 5-fold CV.

For real data, the accuracy of genomic prediction was evaluated as *r*(EBV, PBV) for BLUP and ssGBLUP methods and *r*(*y*_*c*_, PBV) for GBLUP method, the correlation between predicted breeding values (PBV) and EBV or *y*_*c*_ in the validation population. In addition, *b*(EBV or *y*_*c*_, PBV), the regression of EBV or *y*_*c*_ on PBV was also calculated to assess the possible inflation or unbiasedness of predictions. It should be noted here that EBV and *y*_*c*_ were calculated based on a 7-trait animal model as described before, while PBV was calculated for validation population by traditional single-trait BLUP, GBLUP, or ssGBLUP assuming the phenotypic values of validation population were missing. For simulated data, the true breeding values (TBV) of all individuals were available, *r*(TBV, PBV) and *b*(TBV, PBV) in the validation population were used to assess the accuracy and unbiasedness.

## Results

### Descriptive Statistics and Estimates of Genetic Parameters

Number of records, birth year of animals, the descriptive statistics, estimated heritabilities and genetic correlations of the 7 body measurement traits and two simulated traits were presented in Tables 1, 2. The average of body measurement traits ranged from 17.98 to 113.52, and the standard deviation (SD) was between 1.03 and 6.30, and the average (SD) of Trait A and Trait B were 2.25 (3.13) and 1.65 (2.51), respectively. The heritabilities of the 7 body measurement traits ranged from 0.27 to 0.50. Across the 7 analyzed traits, the estimated heritability of BL was the highest of 0.5 and CG was the lowest of 0.27. The standard errors (SE) of estimated heritability for all traits were 0.04. The genetic and phenotypic correlations between traits were also show in Table 2. CW and RW have the highest genetic correlation of 0.84 with SE of 0.03, CG and AG have the second highest genetic correlation of 0.75 with SE of 0.06, some traits had negative or positive medium or low genetic correlation (−0.28–0.25), e.g., BL and RW with −0.28, CW and CG with 0.25. There were also very low genetic correlations among 7 body measurement traits, e.g., BL and BH with −0.01, CW and TG with 0.01. The standard error for genetic and phenotypic correlation ranged from 0.06 to 0.11 and 0.01 to 0.02, respectively. In addition, genetic correlation estimates were generally higher than the phenotypic correlations and the values of genetic correlation and phenotypic correlation had consistency, the traits with high genetic correlation had also high phenotypic correlation.

**Table 2:** Heritabilities (diagonal, bold), genetic correlations (above diagonal), and phenotypic correlations (below diagonal) for seven body measurement traits.

**Trait**^**a**^	**BL**	**BH**	**CW**	**RW**	**CG**	**TG**	**AG**
BL	**0.50**	−0.01	0.02	−0.28	0.04	0.20	−0.11
BH	0.10	**0.30**	−0.22	−0.21	0.18	−0.11	0.06
CW	0.05	−0.08	**0.40**	0.84	0.25	0.01	0.15
RW	−0.04	−0.08	0.74	**0.44**	0.13	−0.03	0.20
CG	0.06	0.09	0.18	0.12	**0.27**	0.19	0.75
TG	0.12	0.05	0.06	0.04	0.16	**0.37**	0.20
AG	0.01	0.02	0.16	0.13	0.63	0.14	**0.30**

a *BL, body length; BH, body height; CW, chest width; RW, rump width; CG, chest girth; TG, tube girth; AG, abdominal girth*.

### Accuracy and Unbiasedness Using Single-Step Strategy and GBLUP Methods on Yorkshire Data

Table 3 presented the accuracy and unbiasedness of genomic prediction from 20 replicates of 5-fold cross validation on the 7 body measurement traits by applying single-step strategy. In a fold of each 5-fold CV, about 100 genotyped individuals were randomly selected as validation populations, the other individuals (about 400) were genotyped reference population for genome prediction, and all non-genotyped animals were added to the analysis for BLUP and ssGBLUP methods. In all scenarios, because of the small size of reference population, GBLUP method generally yielded lower prediction accuracies than BLUP and ssGBLUP methods, the accuracies ranged from 0.32 for BL and CG to 0.504 for RW, the average unbiasedness of prediction was 1.033, however the average SD of unbiasedness was large as 0.278. For ssGBLUP, the average accuracy and unbiasedness of prediction were 0.693 and 0.941, respectively, and the highest accuracy was 0.785 for CG, however the lowest accuracy was 0.543 for BL. The regression coefficients of prediction were also shown in Table 3 which ranged from 0.834 for BL to 1.026 for CG. In 20 replications of 5-fold CV, the SD of prediction accuracy for 7 body measurement traits were all under 0.08, indicating the accuracy in different folds was similar, while the SD of regression coefficients were 0.148 averagely, implying the unbiasedness in different folds changed dramatically.

**Table 3:** The numbers of genotyped animals and the accuracy and unbiasedness of prediction for different traits in 20 replicates of 5-fold cross-validation.

**Trait**^**a**^	**Genotyped animals**	**BLUP**		**GBLUP**		**ssGBLUP**	
		**Accuracy**	**Unbiasedness**	**Accuracy**	**Unbiasedness**	**Accuracy**	**Unbiasedness**
BL	589	0.531 ± 0.058	0.845 ± 0.154	0.320 ± 0.080	0.983 ± 0.341	0.543 ± 0.057	0.834 ± 0.154
BH	589	0.680 ± 0.053	0.873 ± 0.153	0.336 ± 0.075	1.073 ± 0.345	0.689 ± 0.051	0.875 ± 0.156
CW	589	0.677 ± 0.050	0.946 ± 0.159	0.369 ± 0.074	1.010 ± 0.267	0.684 ± 0.079	0.895 ± 0.165
RW	589	0.649 ± 0.055	0.983 ± 0.146	0.504 ± 0.066	0.991 ± 0.157	0.655 ± 0.058	0.971 ± 0.136
CG	589	0.775 ± 0.019	1.048 ± 0.166	0.320 ± 0.069	0.999 ± 0.237	0.785 ± 0.021	1.026 ± 0.169
TG	589	0.732 ± 0.038	1.000 ± 0.094	0.412 ± 0.079	1.050 ± 0.324	0.743 ± 0.036	0.986 ± 0.086
AG	518	0.743 ± 0.050	1.009 ± 0.166	0.336 ± 0.037	1.125 ± 0.272	0.756 ± 0.048	1.002 ± 0.172

a *BL, body length; BH, body height; CW, chest width; RW, rump width; CG, chest girth; TG, tube girth; AG, abdominal girth*.

The accuracy and unbiasedness of traditional BLUP with the same reference and validation population as ssGBLUP was also demonstrated in Table 3. Generally, ssGBLUP provided higher accuracies of predictions than traditional BLUP in all 7 body measurement traits, while the improvement was only 1% on average. The highest increase was 0.013 for AG and the least increase was 0.006 for RW. On the other hand, traditional BLUP generated comparable bias of prediction with ssGBLUP in most traits, the largest difference was for trait of CW, the regression coefficient from BLUP and ssGBLUP was 0.946 and 0.895, respectively. Although the prediction accuracies of ssGBLUP were higher than traditional BLUP, the scope of increase was small, which could be due to small number of genotyped animals.

### The Comparison of Two-Trait Model With Single-Trait Model on Yorkshire Data

In order to evaluate the efficiency of multi-trait model on the improvement of genomic prediction, two-trait ssGBLUP model was compared with single-trait ssGBLUP model. Meanwhile, genetic correlations of three different gradients (high, medium and low) were taken in account in two-trait ssGBLUP to evaluate the impact of genetic correlations on the genomic prediction e.g., high, medium and low genetic correlations of CW with RW (0.84), CG (0.25) and TG (0.01), and three gradients genetic correlations of 0.75 (CG), 0.20 (RW) and 0.06 (BH) with AG. Compared to single-trait model, as shown in Table 4, the accuracies of genomic prediction for CW from two-trait ssGBLUP were increased from 0.684 (Table 3) to 0.703 and 0.697 in the scenarios of high and medium correlations but slightly decreased to 0.676 in low genetic correlation. The gain on accuracy was 2% in situation with high genetic correlation, 1.3% in medium genetic correlation. In addition, the standard deviation of the accuracy in 20 replicates of 5-fold cross validation was all decreased to 0.045, 0.051, and 0.056 from 0.079. On the other hand, the bias of genomic prediction using two-trait model was decreased as well, the regression coefficients were 0.970, 0.953, and 0.959 in high, medium and low genetic correlations, while it was 0.895 in single-trait model. Moreover, the standard deviation of the regression coefficients was also decreased to 0.081, 0.105, and 0.079 from 0.165.

**Table 4:** The accuracy and unbiasedness of genomic prediction of two-trait ssGBLUP in scenarios of different gradients of genetic correlation in 20 replicates of 5-fold cross-validation.

**Genetic correlation**^**b**^	**Trait1**^a^		**Trait2**^a^	
	**Accuracy**	**Unbiasedness**	**Accuracy**	**Unbiasedness**
CW-RW (0.84)	0.703 ± 0.045	0.970 ± 0.081	0.670 ± 0.036	1.009 ± 0.103
CW-CG (0.25)	0.697 ± 0.051	0.953 ± 0.105	0.786 ± 0.019	1.022 ± 0.166
CW-TG (0.01)	0.676 ± 0.056	0.959 ± 0.079	0.736 ± 0.037	0.989 ± 0.085
AG-CG (0.75)	0.760 ± 0.046	1.006 ± 0.088	0.791 ± 0.035	1.026 ± 0.088
AG-RW (0.20)	0.758 ± 0.047	1.002 ± 0.145	0.658 ± 0.062	0.982 ± 0.142
AG-BH (0.06)	0.756 ± 0.044	1.001 ± 0.168	0.688 ± 0.050	0.901 ± 0.158

a *Trait1 were the traits on the left side of the horizontal line, Trait2 were the traits on the right side of the horizontal line; CW, chest width; RW, rump width; CG, chest girth; TG, tube girth; AG, abdominal girth; BH, body height*.

b *Genetic correlation between traits (in parentheses)*.

Similarly, the superiority of two-trait model was also demonstrated on AG. However, only tiny improvement on accuracy was obtained, the accuracies of genomic prediction in three gradient genetic correlations were nearly same and only slightly higher than the those in single-trait model. Likewise, there was no improvement on unbiasedness of genomic prediction from two-trait model as the regression coefficients with 1.002 from single-trait model was already very close to 1.0.

**Figure 1:**
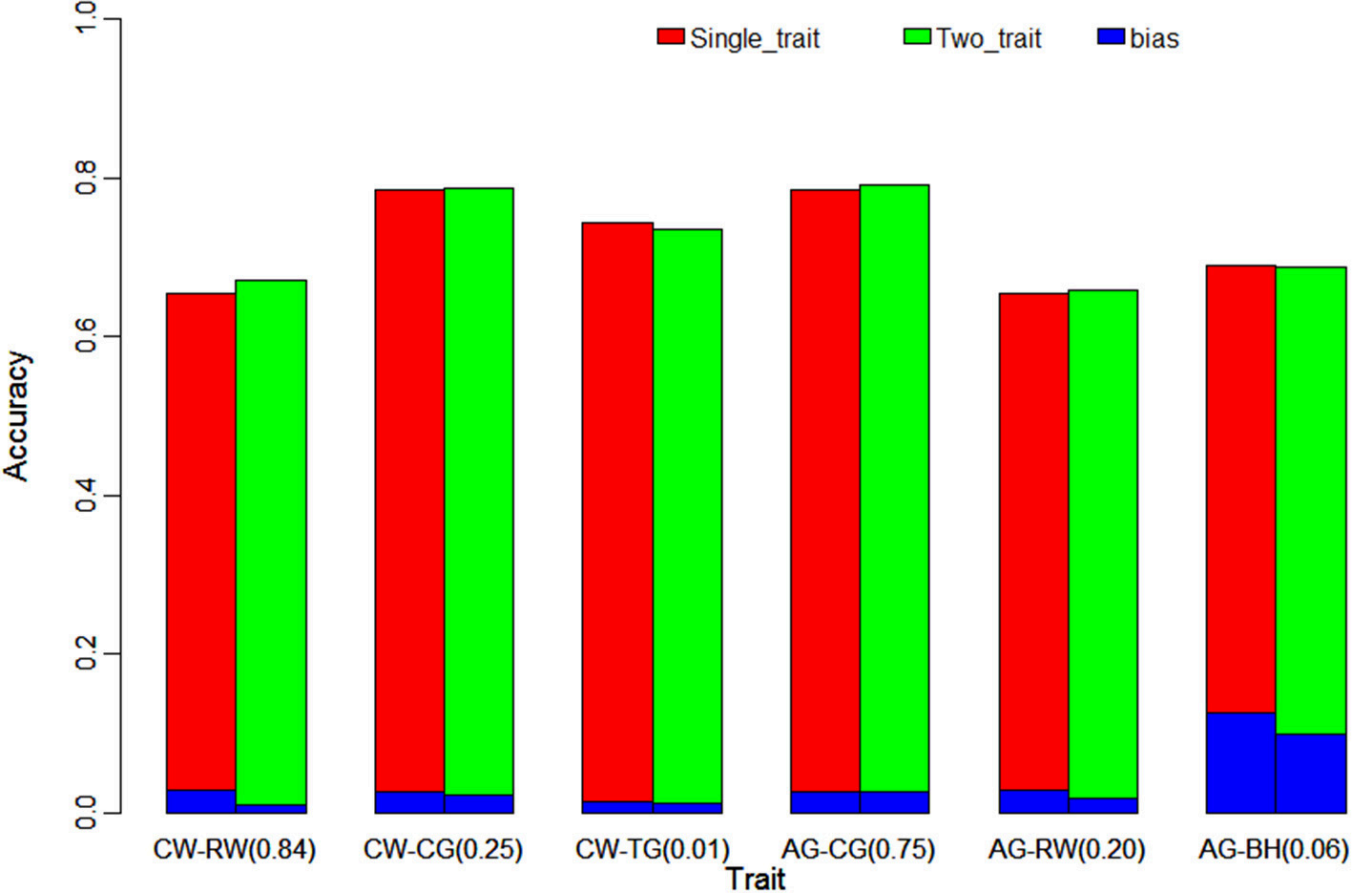
Comparison the accuracy and bias of single-trait ssGBLUP and two-trait ssGBLUP for traits with different genetic correlation (in parentheses). The figure indicates the tiny improvement on accuracy of genomic prediction using two-trait ssGBLUP with different gradients of genetic correlation. CW, chest width; RW, rump width; CG, chest girth; TG, tube girth; AG, abdominal girth; BH, body height.

Table 4 and Figure 1 further indicated the outperformance of two-trait ssGBLUP model. As similar as its performance on trait of CW and AG, in the scenarios of high (0.84) and medium (0.25) genetic correlations for RW and CG with CW, the gains on accuracy were 1.5 and 0.1%, respectively. Likewise, in low genetic correlation (0.01) for TG, the prediction accuracy was slightly decreased from 0.743 to 0.736. In addition, the standard deviation of the accuracy in 20 replicates of 5-fold cross validation was decreased to 0.036 and 0.019 from 0.058 and 0.021 for RW and CG, but no difference for TG. The prediction bias of two-trait model was decreased as well, the regression coefficients were 1.009, 1.022, and 0.989 in high, medium and low genetic correlations, lower than the corresponding 0.971, 1.026, and 0.986 in single-trait model. In addition, the standard deviation of the regression coefficients was also decreased to 0.103, 0.166, and 0.085 from 0.136, 0.169, and 0.086.

The same trend was shown in Figure 1 for high, medium and low genetic correlations of AG with CG (0.75), RW (0.20) and BH (0.06), the improvement in the accuracy of two-trait ssGBLUP over single-trait ssGBLUP was consistently increased with the increase of the genetic correlation from medium to high. Moreover, the accuracy was also decreased in low genetic correlation for BH. Similarly, for RW, it had high correlation (0.84) with CW and medium correlation (0.20) with AG, the gain on its accuracy was 1.5 and 0.3% in these two situations, respectively. The bias of genomic prediction using two-trait model was decreased as well, the regression coefficients were 1.009 and 0.982 in high and medium genetic correlations, while it was 0.971 in single-trait model. The same trend was also for CG which was high correlated with AG (0.75) and medium correlated with CW (0.25) as shown in Table 4 and Figure 1.

### Accuracy and Unbiasedness With Different Genotyped Reference Population Sizes on Simulated Data

**Figure 2:**
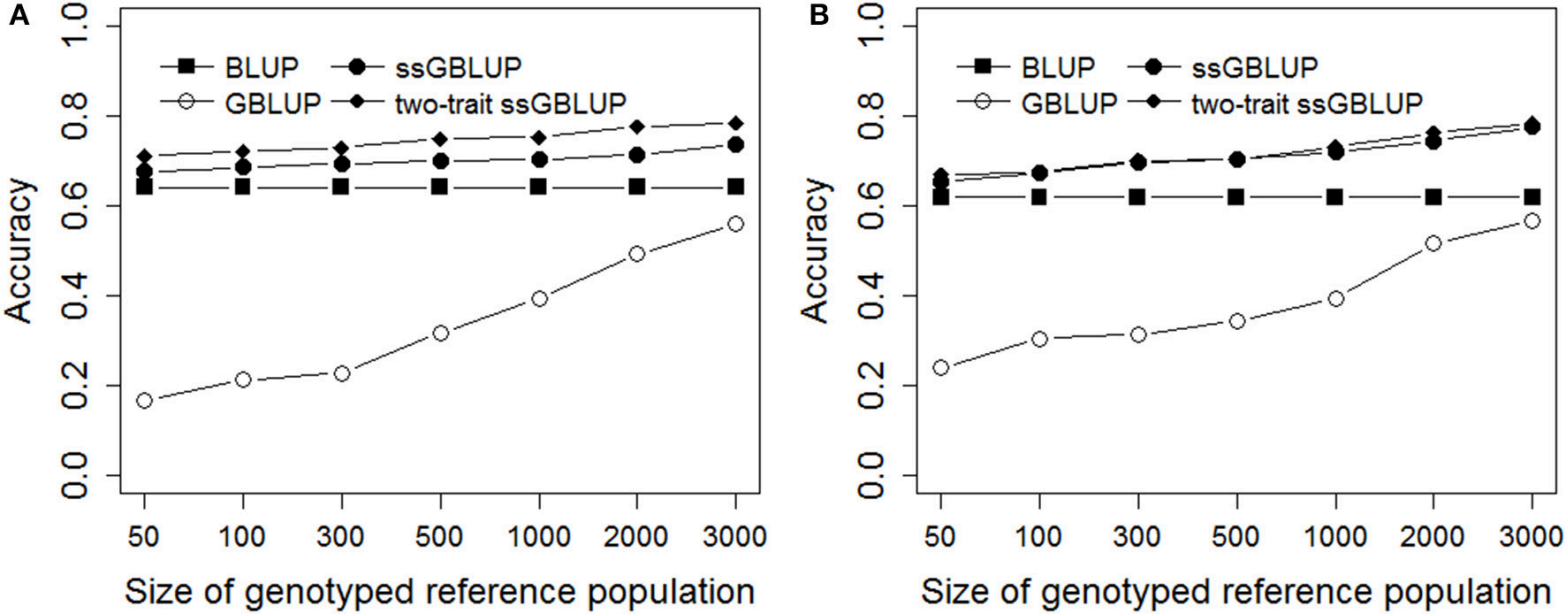
Accuracies with different genotyped reference population sizes on simulated data. The figure indicates the accuracy was improved with increasing the genotyped reference population size. However, the increase was small when the genotyped reference population size was <500 for single-step methods. The heritabilities of Trait A **(A)** and Trait B **(B)** were 0.1 and 0.3.

As shown in Figure 2, the same validation population was predicted through different genotyped reference population sizes using BLUP, GBLUP, ssGBLUP, and two-trait ssGBLUP methods. The accuracies of prediction were low for GBLUP compared with other three methods in all scenarios, while the accuracy of genomic prediction from GBLUP was rapidly increased with increasing the reference population size, especially when the reference population size was enlarged over 500. The accuracy of traditional BLUP with the same reference and validation population as ssGBLUP was also shown in Figure 2. Generally, ssGBLUP provided higher accuracies of predictions than traditional BLUP in different genotyped reference population sizes for Trait A and Trait B, however, the increase was tiny especially in Trait A with low heritability of 0.1 when the genotyped reference population size was below 500, this was consistent with the results of real pig data in this study. In all scenarios, two-trait ssGBLUP produced the highest accuracy for Trait A and Trait B with high genetic correlation of 0.7, but the scope of improvement was low for Trait B with heritability of 0.3.

**Figure 3:**
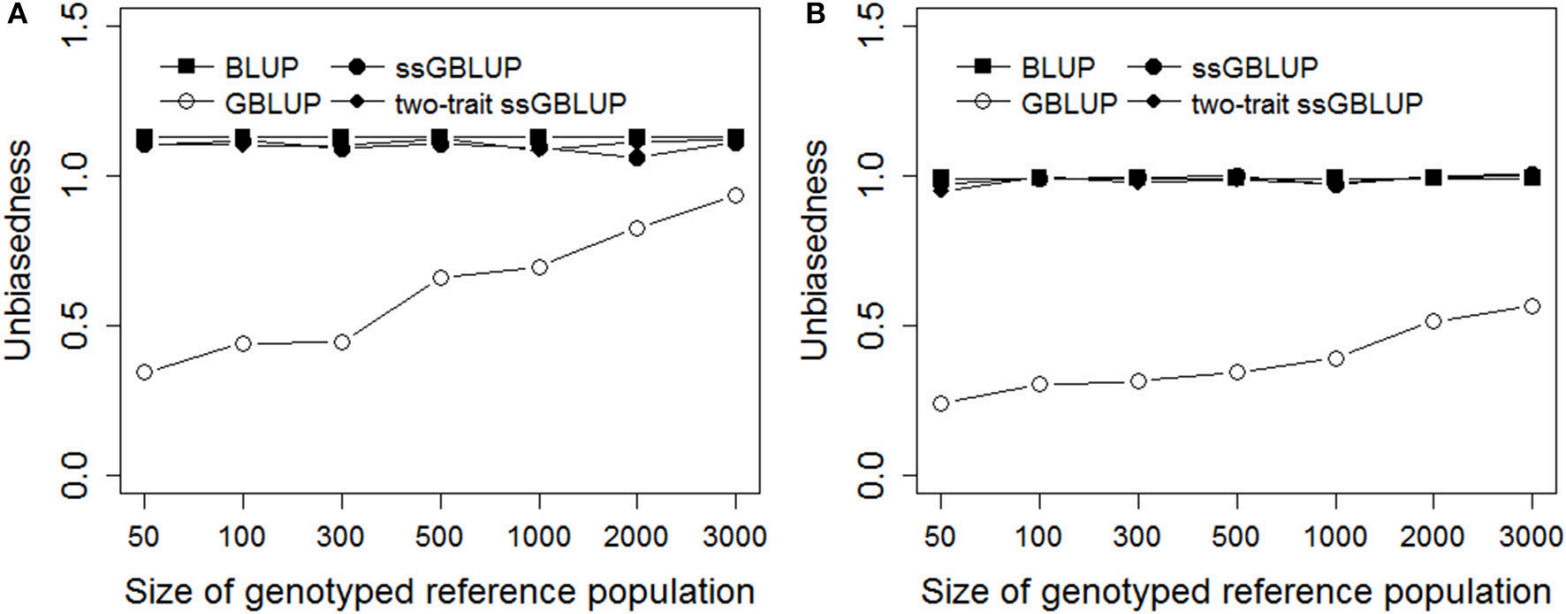
Unbiasedness with different genotyped reference population sizes on simulated data. The figure indicates the regression coefficient was improved with increasing the genotyped reference population size in GBLUP and there was no obvious difference in unbiasedness for Trait B when using BLUP, ssGBLUP, and two-trait ssGBLUP methods. The heritabilities of Trait A **(A)** and Trait B **(B)** were 0.1 and 0.3.

Figure 3 presented the unbiasedness of genomic prediction with different genotyped reference population sizes in Trait A and Trait B. Obviously, the regression coefficients of GBLUP were rapidly increased close to 1.0 with increasing the genotyped reference population size, and the magnitude of the increase was even larger for Trait A which with low heritability of 0.1 than Trait B, the regression coefficient was close to 1 when the genotyped reference population reached 3,000 for Trait A. Furthermore, the biases of genomic prediction for BLUP were a bit larger compared with ssGBLUP and two-trati ssGBLUP methods. However, there was no obvious difference in unbiasedness for Trait B when using BLUP, ssGBLUP and two-trait ssGBLUP methods which were all very close to 1.

## Discussion

In this study, the estimated heritabilities of 7 body measurement traits were medium to high ranging from 0.27 to 0.50. The estimates of heritability of body length (BL) and body height (BH) were consistent with the study by Do et al. (2014) where the estimated heritabilities of BL and BH ranged from 0.331 to 0.559 at the ages of 70, 145, and 180 days, but the estimates of heritability of BL in our study was higher than those reported by Nikkila et al. (2013), where the estimates of heritability of BL was 0.29 in commercial gilts. Zhou et al. (2016) reported that heritabilities were 0.34 and 0.25 for BL, 0.05 and 0.25 for BH, 0.30 and 0.27 for CG at ages of 210 and 240 days in Chinese indigenous Laiwu pig. The difference in heritability estimates may be due to population structure, environmental factors, etc.

The genetic correlations of 7 body measurement traits ranged from −0.28 to 0.84, these traits were highly, medium or low correlated. Usually, the medium or highly correlated traits was more reasonable for multiple-trait BLUP model, while in the estimation of breeding values in this study, two or three highly correlated trait model performed as similar as the 7-trait model (data not shown). The genetic correlation between CW and RW was lower than the value of 0.56 reported by Kutwana et al. (2015). However, research on other livestock, the genetic correlations between BL and CG were higher in sheep which were 0.52 and 0.57 (Janssens and Vandepitte, 2004; Jafari et al., 2014). In cattle, Kolkman et al. (2010) reported that the genetic correlations between BL and RW was close to 0 which was −0.28 in our study. These results imply that genetic correlations among different livestock are quite different. In addition, that both genetic and phenotypic correlation coefficients were of the same sign and magnitude, Roff (1996) reported that the genetically and environmentally (residual) correlations are likely to share the same pattern.

In the single-step strategy, the construction of H matrix involves several parameters and some of them were shown to influence the accuracy and bias of prediction (Christensen et al., 2012; Koivula et al., 2015; Song et al., 2017). In this study, to avoid singularity problems, **w** was set to 0.95 in each analysis as other studies (Aguilar et al., 2010; Lourenco et al., 2014). However, in Danish Landrace and Yorkshire populations, Guo et al. (2015) reported the weight value of 0.5 was fit for three traits, and 0.25 was found to be an ideal value for the Danish Duroc population (Christensen et al., 2012). Generally, the markers may not explain all genetic variance, the weighting factor w is the proportion of genetic variance not captured by markers which is population and trait specific, an optimal weighting factor on A matrix may best use both markers and pedigree information. In addition, in ssGBLUP, the scales of A and G matrix may differ, the adjustment factors (β and α) was used to adjust G matrix to be consistent with scale as A matrix, the commonly used adjustment method was reported by Christensen et al. (2012) as shown in section Single-Trait ssGBLUP of Statistical models. It was reported that the adjusted G resulted in better performance of ssGBLUP than the original G in which the estimates of β and α were 0.859 and 0.298, respectively (Christensen et al., 2012). Therefore, in this study, we used adjusted G to make the scale of the G matrix to be comparable with that of the A matrix.

We investigated the genomic evaluation of body measurement traits of Yorkshire by applying single-step strategy. In this study, the accuracies of prediction ranged from 0.543 to 0.785 with a 5-fold cross-validation, in order to take advantage of all information, all non-genotyped animals were added to construct H matrix in ssGBLUP. As both the genomic and pedigree information was utilized by ssGBLUP, previous studies have shown that the ssGBLUP method was superior to both the GBLUP method (Christensen et al., 2012; Gao et al., 2012; Li et al., 2014; Guo et al., 2015; Song et al., 2017) and traditional pedigree-based BLUP (Gao et al., 2012; Koivula et al., 2015), in which only genomic or pedigree information was used. Our findings are consistent with these reports and further proved the superiority of single-step strategy, however, compared to the traditional BLUP, in our study, ssGBLUP only generated tiny higher accuracies with 1% on average increase in 7 body measurement traits and simulated data when the number of genotyped reference animals was small.

The lower improvement of ssGBLUP might possibly attribute from the following reasons. (1) The genotyped reference was not large enough to improve the genomic predictive ability. In real data, about 400 genotyped reference animals could not probably provide more extra information compared to the pedigree information consisting of 5,000 individuals. Similarly, it also deduced the lower accuracy of GBLUP than BLUP in this study. In simulation study, GBLUP also produced lower accuracy than BLUP even the genotyped reference population size reached 3,000, it was still very small compared to non-genotyped individuals of 26,000 utilized by BLUP. Meanwhile, the improvement was also tiny for ssGBLUP compared to BLUP when the genotyped reference population size was small. Lourenco et al. (2014) also reported that GBLUP performed worse than BLUP and ssGBLUP only generated 3% higher accuracy than BLUP for fat percentage in all parities in a relatively small genotyped dairy population; (2) Inappropriate w value may cause the larger bias of ssGBLUP. In constructing H matrix, the weighting factor (w) was the proportion of genetic variance not captured by markers, theoretically, w value depends on the trait, the same w value was assigned in our study, deducing the larger bias of genomic prediction of ssGBLUP than BLUP, e.g., BL and CW. This indicated that in implementation of single-step genomic evaluation, it is better to test different w to find out the optimal parameter for prediction; (3) In our study, the heritabilities for 7 body measurement traits were high from 0.27 to 0.5, which can obtain sufficient accuracy for traditional BLUP method, and improvement from genomic prediction was not large as expected. Other studies also indicated that for low-heritability traits, a very large number of records will be required in the reference population to subsequently achieve high accuracies of GEBV in unphenotyped animals, while for the traits with medium or high heritabiliries the tiny improvement will be achieved when added a small reference population (Goddard and Hayes, 2009; Hayes et al., 2009b), our findings in simulated data are consistent with these reports.

Genetic correlation between traits has been used to improve the statistical power to detect QTL controlling traits of interest (Chesler et al., 2005; Xu et al., 2009). A series of researches of multiple traits have also been carried out in genomic selection and showed that multiple-trait model can improve the accuracy of genomic prediction compared to single-trait model (Calus and Veerkamp, 2011; Jia and Jannink, 2012; Guo et al., 2014). Our findings further proved that two-trait ssGBLUP yielded higher accuracy and lower bias than single-trait ssGBLUP.

Our results also indicated that the performance of two-trait ssGBLUP was related with the strength of genetic correlation. The stronger the genetic correlation is, the higher improvement on the accuracy of genomic prediction, e.g., in the scenarios of high and medium genetic correlations, the maximum gain on accuracy was 2 and 2.6% for trait of CW compared to single-trait ssGBLUP and traditional BLUP, while the gain was decreased in the low genetic correlation. The same trend was illustrated on other body measurement traits, the difference was the scope of the improvement. e.g., compared to CW, only tiny improvement on accuracy was obtained for trait of AG in both high and medium correlations. Generally, in our study, as the heritabilities for 7 body measurement traits highly ranged from 0.27 to 0.5, the improvement on accuracy from two-trait genomic model was not as large as on the low-heritability trait. As shown in the results of simulated data, the Trait A with heritability of 0.1 produced larger gain on accuracy than Trait B with heritability of 0.3 when using two-trait ssGBLUP method (Figure 3). This was consistent with other previous reports (Calus and Veerkamp, 2011; Jia and Jannink, 2012; Guo et al., 2014).

However, in our study, in low genetic correlation (e.g., 0.01 and 0.06), the accuracies of genomic prediction were lower than that of separate single trait analysis, which was also reported by Jia and Jannink (2012) and Wang et al. (2017), The reason may be that, in this case, sampling from multiple trait model leads to nonzero estimates of correlation, which then leads to error information sharing across traits, deducing the lower accuracy of two-trait model. Therefore, for one trait correlated with several traits, the largest improvement of the accuracy and unbiasedness of genomic prediction on this target trait can be obtained by choosing one trait with highest genetic correlation with the target trait through two-trait ssGBLUP model. However, only two-trait model was investigated in this study, multiple-trait model is worth exploring in the future, however, it should be noted that much more computational demand will be increased as more random variance components needed to be estimated, and the equation is also difficult to converge. Furthermore, the traditional animal model assumes the linear relationship between the observations breeding values and the covariates, however, in practice, the nonlinear relationship is more common. In theory, the single-step genomic prediction using information from both genotyped and non-genotyped animals could be extended to generalized linear model or Bayesian model (Fernando et al. (2014), while the higher computational demand and the efficiency of genomic prediction in practice should be taken into account in the further investigation.

## Conclusion

In this study, we used different single-step strategies and GBLUP method to investigate the efficiency of genomic prediction on body measurement traits in Yorkshire and simulated data. We compared the prediction accuracy of a traditional pedigree-based method (BLUP) with the GBLUP and single-step genomic BLUP (ssGBLUP) through 20 replicates of 5-fold cross-validation (CV) in pig data. Meanwhile, we investigated the impact of genotyped reference population size on the accuracy of genomic prediction through simulation study. In addition, we also compared the performance of two-trait ssGBLUP and single-trait ssGBLUP for the traits with different gradients of genetic correlation. Our results indicated that ssGBLUP generally generated higher prediction accuracy than traditional BLUP, the improvement was lower than expected mainly due to the small number of genotyped animals. Our results also demonstrated that the accuracies of genomic prediction can be further improved by implementing two-trait ssGBLUP model.

## Data Availability

The simulated data and 7 body measurement traits data of Yorkshire supporting the conclusions of this article are available from the Figshare: https://figshare.com/articles/single-step_strategies/7434203.

## Author Contributions

XD and QZ designed and supervised the study. HS conducted the statistical analyses and interpretation of results. JZ conceived the experimental traits and sample preparation for DNA genotyping. HS simulated the data and wrote the manuscript, which was critically remarks by XD and QZ. All authors read and approved the manuscript.

### Conflict of Interest Statement

The authors declare that the research was conducted in the absence of any commercial or financial relationships that could be construed as a potential conflict of interest.

## References

[B1] Aguilar I. Misztal I. Johnson D. L. Legarra A. Tsuruta S. Lawlor T. J. (2010). Hot topic: A unified approach to utilize phenotypic, full pedigree, and genomic information for genetic evaluation of Holstein final score. J. Dairy Sci. 93, 743–752. 10.3168/jds.2009-2730 20105546

[B2] Aguilar I. Misztal I. Tsuruta S. Wiggans G. R. Lawlor T. J. (2011). Multiple trait genomic evaluation of conception rate in Holsteins. J. Dairy Sci. 94, 2621–2624. 10.3168/jds.2010-3893 21524554

[B3] Browning B. L. Browning S. R. (2009). A unified approach to genotype imputation and haplotype-phase inference for large data sets of trios and unrelated individuals. Am. J. Hum. Genet. 84, 210–223. 10.1016/j.ajhg.2009.01.005 19200528 PMC2668004

[B4] Calus M. P. L. Veerkamp R. F. (2011). Accuracy of multi-trait genomic selection using different methods. Genet. Sel. Evol. 43:26. 10.1186/1297-9686-43-26 21729282 PMC3146811

[B5] Chen C. Y. Misztal I. Aguilar I. Tsuruta S. Meuwissen T. H. Aggrey S. E.. (2011). Genome-wide marker-assisted selection combining all pedigree phenotypic information with genotypic data in one step: an example using broiler chickens. J. Anim. Sci. 89, 23–28. 10.2527/jas.2010-3071 20889689

[B6] Chesler E. J. Lu L. Shou S. M. Qu Y. H. Gu J. Wang J. T.. (2005). Complex trait analysis of gene expression uncovers polygenic and pleiotropic networks that modulate nervous system function. Nat. Genet. 37, 233–242. 10.1038/ng1518 15711545

[B7] Christensen O. F. Lund M. S. (2010). Genomic prediction when some animals are not genotyped. Genet. Sel. Evol. 42:2 10.1186/1297-9686-42-2 20105297 PMC2834608

[B8] Christensen O. F. Madsen P. Nielsen B. Ostersen T. Su G. (2012). Single-step methods for genomic evaluation in pigs. Animal 6, 1565–1571. 10.1017/S1751731112000742 22717310

[B9] Do C. Park C. Wasana N. Choi J. Park S. B. Kim S. (2014). Genetic and phenotypic relationships of live body measurement traits and carcass traits in crossbred pigs of Korea. Korean J. Agric. Sci. 41, 229–236. 10.7744/cnujas.2014.41.3.229

[B10] Enevoldsen C. Kristensen T. (1997). Estimation of body weight from body size measurements and body condition scores in dairy cows. J. Dairy Sci. 80, 1988–1995. 10.3168/jds.S0022-0302(97)76142-3 9313139

[B11] Fernando R. L. Dekkers J. C. M. Garrick D. J. (2014). A class of Bayesian methods to combine large numbers of genotyped and non-genotyped animals for whole-genome analyses. Genet. Sel. Evol. 46:50. 10.1186/1297-9686-46-50 25253441 PMC4262255

[B12] Forni S. Aguilar I. Misztal I. (2011). Different genomic relationship matrices for single-step analysis using phenotypic, pedigree and genomic information. Genet. Sel. Evol. 43:1. 10.1186/1297-9686-43-1 21208445 PMC3022661

[B13] Gao H. D. Christensen O. F. Madsen P. Nielsen U. S. Zhang Y. Lund M. S.. (2012). Comparison on genomic predictions using three GBLUP methods and two single-step blending methods in the Nordic Holstein population. Genet. Sel. Evol. 44:8. 10.1186/1297-9686-44-8 22455934 PMC3400441

[B14] Goddard M. E. Hayes B. J. (2009). Mapping genes for complex traits in domestic animals and their use in breeding programmes. Nat. Rev. Genet. 10, 381–391. 10.1038/nrg2575 19448663

[B15] Guo G. Zhao F. P. Wang Y. C. Zhang Y. Du L. X. Su G. S. (2014). Comparison of single-trait and multiple-trait genomic prediction models. BMC Genet. 15:30. 10.1186/1471-2156-15-30 24593261 PMC3975852

[B16] Guo X. Christensen O. F. Ostersen T. Wang Y. Lund M. S. Su G. (2015). Improving genetic evaluation of litter size and piglet mortality for both genotyped and nongenotyped individuals using a single-step method. J. Anim. Sci. 93, 503–512. 10.2527/jas.2014-8331 25549983

[B17] Habier D. Fernando R. L. Kizilkaya K. Garrick D. J. (2011). Extension of the bayesian alphabet for genomic selection. BMC Bioinformatics 12:186. 10.1186/1471-2105-12-186 21605355 PMC3144464

[B18] Hayes B. J. Bowman P. J. Chamberlain A. C. Verbyla K. Goddard M. E. (2009a). Accuracy of genomic breeding values in multi-breed dairy cattle populations. Genet. Sel. Evol. 41:51. 10.1186/1297-9686-41-51 19930712 PMC2791750

[B19] Hayes B. J. Bowman P. J. Chamberlain A. J. Goddard M. E. (2009b). Invited review: genomic selection in dairy cattle: progress and challenges (vol 92, pg 433, 2009). J. Dairy Sci. 92:1313 10.3168/jds.2008-1646 19164653

[B20] Jafari S. Hashemi A. Darvishzadeh R. Manafiazar G. (2014). Genetic parameters of live body weight, body measurements, greasy fleece weight, and reproduction traits in Makuie sheep breed. Span. J. Agric. Res. 12, 653–663. 10.5424/sjar/2014123-4564

[B21] Janssens S. Vandepitte W. (2004). Genetic parameters for body measurements and linear type traits in Belgian Bleu du Maine, Suffolk and Texel sheep. Small Ruminant Res. 54, 13–24. 10.1016/j.smallrumres.2003.10.008

[B22] Jia Y. Jannink J. L. (2012). Multiple-trait genomic selection methods increase genetic value prediction accuracy. Genetics 192, 1513–1522. 10.1534/genetics.112.144246 23086217 PMC3512156

[B23] Koivula M. Stranden I. Poso J. Aamand G. P. Mantysaari E. A. (2015). Single-step genomic evaluation using multitrait random regression model and test-day data. J. Dairy Sci. 98, 2775–2784. 10.3168/jds.2014-8975 25660739

[B24] Kolkman I. Opsomer G. Aerts S. Hoflack G. Laevens H. Lips D. (2010). Analysis of body measurements of newborn purebred Belgian Blue calves. Animal 4, 661–671. 10.1017/S1751731109991558 22444118

[B25] Kutwana H. W. Gxasheka M. Tyasi T. L. (2015). Body weight and morphological traits of large white and Kolbroek pig breeds. Int. J. Adv. Res. 3, 105–109. Available online at: http://www.journalijar.com/uploads/50_IJAR-6862.pdf

[B26] Legarra A. Aguilar I. Misztal I. (2009). A relationship matrix including full pedigree and genomic information. J. Dairy Sci. 92, 4656–4663. 10.3168/jds.2009-2061 19700729

[B27] Li X. J. Wang S. Huang J. Li L. Y. Zhang Q. Ding X. D. (2014). Improving the accuracy of genomic prediction in Chinese Holstein cattle by using one-step blending. Genet. Sel. Evol. 46:4. 10.1186/s12711-014-0066-4 25315995 PMC4196050

[B28] Lourenco D. A. L. Misztal I. Tsuruta S. Aguilar I. Ezra E. Ron M.. (2014). Methods for genomic evaluation of a relatively small genotyped dairy population and effect of genotyped cow information in multiparity analyses. J. Dairy Sci. 97, 1742–1752. 10.3168/jds.2013-6916 24472123

[B29] Meuwissen T. H. E. Hayes B. J. Goddard M. E. (2001). Prediction of total genetic value using genome-wide dense marker maps. Genetics 157, 1819–1829. Available online at: http://www.genetics.org/content/genetics/157/4/1819.full.pdf 11290733 10.1093/genetics/157.4.1819 PMC1461589

[B30] Mutua F. K. Dewey C. E. Arimi S. M. Schelling E. Ogara W. O. (2011). Prediction of live body weight using length and girth measurements for pigs in rural Western Kenya. J. Swine Health Prod. 19, 26–33. Available online at: https://cgspace.cgiar.org/handle/10568/3087

[B31] Nikkila M. T. Stalder K. J. Mote B. E. Rothschild M. F. Gunsett F. C. Johnson A. K.. (2013). Genetic parameters for growth, body composition, and structural soundness traits in commercial gilts. J. Anim. Sci. 91, 2034–2046. 10.2527/jas.2012-5722 23408822

[B32] Roff D. A. (1996). The evolution of genetic correlations: an analysis of patterns. Evolution 50, 1392–1403. 10.1111/j.1558-5646.1996.tb03913.x 28565723

[B33] Simeone R. Misztal I. Aguilar I. Vitezica Z. G. (2012). Evaluation of a multi-line broiler chicken population using a single-step genomic evaluation procedure. J. Anim. Breed. Genet. 129, 3–10. 10.1111/j.1439-0388.2011.00939.x 22225579

[B34] Song H. Zhang J. Jiang Y. Gao H. Tang S. Mi S.. (2017). Genomic prediction for growth and reproduction traits in pig using an admixed reference population. J. Anim. Sci. 95, 3415–3424. 10.2527/jas2017.1656 28805914

[B35] Thiruvenkadan A. K. (2005). Determination of best-fitted regression model for estimation of body weight in Kanni Adu kids under farmer's management system. Livestock Res. Rural Dev. 17:85 Available online at: http://www.lrrd.org/lrrd17/7/thir17085.htm

[B36] VanRaden P. M. (2008). Efficient methods to compute genomic predictions. J. Dairy Sci. 91, 4414–4423. 10.3168/jds.2007-0980 18946147

[B37] Wang C. Li X. Qian R. Su G. Zhang Q. Ding X. (2017). Bayesian methods for jointly estimating genomic breeding values of one continuous and one threshold trait. PLoS ONE 12:e0175448. 10.1371/journal.pone.0175448 28410429 PMC5391971

[B38] Xu C. W. Wang X. F. Li Z. K. Xu S. Z. (2009). Mapping QTL for multiple traits using Bayesian statistics. Genet. Res. 91, 23–37. 10.1017/S0016672308009956 19220929

[B39] Zhang Z. Li X. J. Ding X. D. Li J. Q. Zhang Q. (2015). GPOPSIM: a simulation tool for whole-genome genetic data. BMC Genet. 16:4. 10.1186/s12863-015-0173-4 25652552 PMC4328615

[B40] Zhou L. S. Ji J. X. Peng S. Zhang Z. Fang S. M. Li L.. (2016). A GWA study reveals genetic loci for body conformation traits in Chinese Laiwu pigs and its implications for human BMI. Mamm. Genome 27, 610–621. 10.1007/s00335-016-9657-4 27473603

